# Evaluating the Clinical Validity and Reliability of Artificial Intelligence-Enabled Diagnostic Tools in Neuropsychiatric Disorders

**DOI:** 10.7759/cureus.71651

**Published:** 2024-10-16

**Authors:** Satneet Singh, Jade L Gambill, Mary Attalla, Rida Fatima, Amna R Gill, Humza F Siddiqui

**Affiliations:** 1 Psychiatry, Hampshire and Isle of Wight Healthcare NHS Foundation Trust, Southampton, GBR; 2 Neuroscience, Parker University, Dallas, USA; 3 Medicine, Saba University School of Medicine, The Bottom, NLD; 4 Mental Health, Cwm Taf Morgannwg University Health Board, Pontyclun, GBR; 5 Psychiatry, HSE (Health Service Executive) Ireland, Dublin, IRL; 6 Internal Medicine, Jinnah Postgraduate Medical Centre, Karachi, PAK

**Keywords:** alzheimer disease, artificial intelligence (ai), cognitive impairment, depression, machine learning (ml), neuropsychiatric disorders (npds)

## Abstract

Neuropsychiatric disorders (NPDs) pose a substantial burden on the healthcare system. The major challenge in diagnosing NPDs is the subjective assessment by the physician which can lead to inaccurate and delayed diagnosis. Recent studies have depicted that the integration of artificial intelligence (AI) in neuropsychiatry could potentially revolutionize the field by precisely diagnosing complex neurological and mental health disorders in a timely fashion and providing individualized management strategies. In this narrative review, the authors have examined the current status of AI tools in assessing neuropsychiatric disorders and evaluated their validity and reliability in the existing literature. The analysis of various datasets including MRI scans, EEG, facial expressions, social media posts, texts, and laboratory samples in the accurate diagnosis of neuropsychiatric conditions using machine learning has been profoundly explored in this article. The recent trials and tribulations in various neuropsychiatric disorders encouraging future scope in the utility and application of AI have been discussed. Overall machine learning has proved to be feasible and applicable in the field of neuropsychiatry and it is about time that research translates to clinical settings for favorable patient outcomes. Future trials should focus on presenting higher quality evidence for superior adaptability and establish guidelines for healthcare providers to maintain standards.

## Introduction and background

It is estimated that one in eight suffer from a neuropsychiatric disorder (NPD) worldwide, according to the World Health Organization (WHO) [1]. NPDs may impair normal thinking, mood, or behavior and can also place a severe burden on public health as they significantly increase the risk of disability, pain, mortality, or loss of autonomy [2]. Among the several classes of mental illness that exist, early detection and accurate diagnostic modalities are going to be more and more crucial. Diagnosis of NPDs using existing modalities is largely based on physician-patient questionnaires, wherein the inherent subjectiveness can result in a biased and inaccurate measure for specific symptoms or could even be non-efficient causing detrimental patient outcomes. For instance, depressive disorder and generalized anxiety disorder (GAD) have a wide variety of overlapping symptoms intermittent in time leading to incorrect diagnosis by psychiatrists as either disease primarily or a comorbid condition [1,2].

Artificial intelligence (AI) is an exciting and rapidly growing field of computer science focused on enabling computers to accomplish jobs that require human efforts. AI refers to a series of transformative technologies and constitutes subfields including supervised learning, unsupervised learning, and deep learning [3]. Machine learning (ML) is a model trained with labeled datasets that output predictions or decisions from the input data and answer expected variables. On the other hand, unsupervised ML works with data to discover patterns or relationships in information for tasks like clustering and dimensionality reduction. Deep learning takes this one step further, with deep neural networks made up of stacked layers that can capture complex relationships and representations. Deep learning was inspired by the structure and functions of the brain to learn independently in AI systems, which enables them to do tasks or adjust accordingly [4]. In all respects, this allows machines to mimic any kind of intelligent activity. Integration of these widespread methods is what becomes a powerful tool for AI in the technology landscape today [3,4].

Over the years, it was discovered that many do not receive a diagnosis in medical and psychiatric realms due to stigma, high burden of achieving treatment, or simply lack of vetted professionals readily available. However, implementing AL technology in electronic health records and medical databases can individualize the diagnostic process. Furthermore, AI tools can be applied to underserved areas for the care of vulnerable populations with NPDs in those regions [5]. Natural language processing (NLP) and learning health systems in AI for neuropsychiatry are moving from the bedside to being able to diagnose, prevent, and treat various mental disorders and illnesses. Because AI can analyze massive databases of patient information far more quickly and accurately than a human, it has the potential to improve diagnostic accuracy by spotting patterns that are hard for humans to notice [6]. AI pattern-recognizing software has been successful in identifying the signs of anxiety and depression using facial expressions, images, emotional chatbots, or even texts on social media platforms. This intriguing data analysis anticipated the rise of depression or anxiety among particular types of demographic groups [7]. The data analysis process includes ML algorithms (MLAs) that can be quite complex but might potentially enable the development of individually personalized treatment options for each patient [8]. A single randomized-controlled trial reported evidence that an AI-generated algorithm named MJN Neuroserveis Seizure Alert System (MJN-SERAS; MJN Neuroserveis, SL, Blanes, Girona, Spain) can recognize potential seizures in patients with refractory focal epilepsy, using electroencephalography (EEG) data [9]. A recent cross-trial study also used MLAs to forecast a six-month remission of depression following problem-solving therapy (PST) [10].

**Figure 1:**
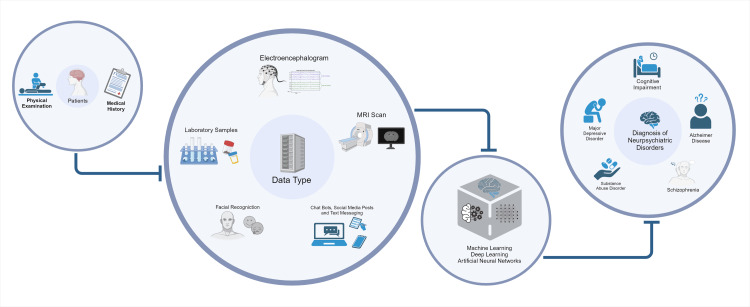
Overview of use of artificial intelligence and machine learning in diagnosing neuropsychiatric disorders. Image Credit: Authors; made using biorender.com

Another study was able to go one step further even by developing a model-agnostic explanation for the sake of clinical decision-making [11]. The input of deep learning is through raw data, i.e., numbers (in any form), images, text and molecular information, or medical signs and natural language. It makes use of multiple layers to process data and output it properly. NLP, a deep learning application in the domain of neuropsychiatry, uses automated means to create representations or “human-like” text and speech [11]. Text analysis of a patient’s medical history using NLPs has emerged as an encouraging tool to evaluate the risk stratification for post-traumatic stress disorder (PTSD) [12]. AI-driven robots are also an advancing development and can produce hopeful results in children with autism spectrum disorders (ASDs) [13]. Kaspar robot (University of Hertfordshire, Hatfield, United Kingdom) has shown some potential to be integrated into educational and therapy interventions that are more efficient in improving social skills among children [14]. Though AI and MLAs have shown exponential growth in their application to medical practice, the goal of this review is not only to introduce deep learning models currently used in clinical settings but also to provide a levelheaded view of predictive validity which is central to decision making. Figure 1 summarizes the use of AI diagnostic tools in NPDs. 

## Review

Clinical validity of AI tools

Clinical validity is a concept in neuropsychiatric diagnosis that refers to the ability of a diagnostic instrument including AI applications to recognize and categorize individuals with particular diseases accurately. It mainly indicates the sensitivity, specificity, positive predictive value, and negative predictive value of a diagnostic modality. Relevance of clinical validity is essential for patient progression, treatment pathway decisions, and the efficacy of healthcare interventions as a whole. For a clinician in the trenches of NPDs, true clinical validity is what makes or breaks an AI tool when it comes to accurate and timely diagnosis.

A study conducted by Fei et al. implemented facial expression recognition by making minor modifications to MobileNet for feature extraction from image samples and using support vector machines (SVM) to classify the extracted features accordingly. Facial expressions depicting various complex emotions from watching the sampled videos were collected from 61 participants, both cognitively impaired and healthy, in a laboratory environment using the proposed system. Emotions were detected by an imitation-related facial expression recognition algorithm employing MobileNet + block_11_add + SVM and a total of 61 pieces of emotion data was collected. Dementia prediction for the testing dataset was performed using an SVM classifier trained with 46 samples of emotion data on this classification label, which enabled the detection of cognitive impairment with a success rate of 73.3% accuracy. It is an inexpensive method that has moderate accuracy in identifying the early stages of cognitive impairment [15].

In a multicenter data study by Kang et al., 14,926 neuropsychiatric subjects were classified into three diagnostic groups including normal cognition, mild cognitive impairment (MCI), or Alzheimer’s disease (AD) based on assessments [16]. They used the artificial neural network algorithm TensorFlow (tensorflow.org) to detect different cognitive states. Kang et al. assessed the predictive performance for the neuropsychiatric stage of logistic regression and neural network using features based on the Seoul Neuropsychological Screening Battery (SNSB) [15]. Their three-layer neural network achieved impressive accuracies of 96.44% (± 0.96%) and 95.89% (± 0.99%) in two-way and three-way classification tasks using a balanced dataset, and 97.51% (± 0.40%) and 97.01% (± 0.54%) using a clinic-based dataset. In the two-way classification (cognitive impairment vs. normal cognition) of cognitive impairment prediction, the accuracies obtained by this algorithm were 96.66% (±0.52%) and 97.23% (±.032%), respectively [16]. The corresponding accuracies in detecting MCI from normal cognition were 96.60 (±0.45%) and 97.05% (±0.38%) respectively. The accuracy of predicting one of the three outcomes (normal cognition, MCI, or AD), was 95.49% (±0.53 %) and 96.34% (±1.03 %). The importance of 46 variables was decided through recursive feature elimination (RFE). The most important features utilized for predicting with Mini-Mental State Examination (MMSE) were orientation to time and memory recall. Despite having only six features, the prediction of cognitive status exceeded in accuracy by 80%. Additionally, as the number of features increased to 10 and above, accuracies of more than 90% were achieved in two-way and three-way classification tasks [16].

Torres-Gaona et al., in another multicenter clinical cohort study, analyzed 50 refractory focal epilepsy patients subjected to video-EEG monitoring [9]. An algorithm to identify pre-ictal and interictal patterns in the EEG data was utilized by a research team. This was implemented through the MJN-SERAS AI algorithm used for data reading and analysis, specifically in bioinformatics. The MJN-SERAS AI algorithm had 94.7% sensitivity (95%CI: 94.67-94.73) and 92.2% specificity (95%CI: 92,17- 92,23) compared with standard performance. The patient-independent model had a false positive rate of 0.55 once every day. In total, this customized AI model showed relatively high sensitivity and lower false positive rate values in the performance evaluation test that could support early detection of a seizure.

One study utilized the social media posts on X (X Corp., Bastrop, Texas, United States) to identify depression using the “Naive Bayes” algorithm, which produced an A-grade receiver operating curve (ROC), area under the curve (AUC) of 0.94 with a precision score of 0.82, and an accuracy of 86%. Meanwhile the fundamental “Bag of Words” approach which is used in NLP and information retrieval yielded distinct advantages, in addition, the unigram-based approach was found to have a significant impact which was not as evident when bigrams were included. It is worthy of note that In NLP the unigram-based approach is very commonly used in language modeling. In this approach, each word is a distinct entity and the frequency of each word is recorded independently with no link to other words [7]. The algorithm Multinominal Naive Bayes (MNB) has been shown to perform better than the SVM algorithm in a study which suggests that the MNB algorithm is more appropriate for tasks where robust precision and predictive power are required [17].

In one study by Singh et al., in an attempt to use the computer to accurately predict human emotions, they used convolutional neural networks (CNN) in combination with adaptive moment estimation (ADAM). They introduced CNN to a facial recognition data set that comprised thousands of images depicting human emotions and later implemented this dataset to identify the emotions of the model participants. The final results showed that the accuracy rate was between 66.4% in training and 67.6% in testing to determine the correct emotion. This shows a promising future for the ability of AI to accurately determine human emotions provided it undergoes rigorous and strategic training [18].

Cook et al. applied NLP and ML to assess suicide risk probability among patients in the psychiatry unit and emergency department with a positive history of suicide attempts [19]. They implemented an ML software that employed the clinical Text Analysis Knowledge Extract System (cTAKES) to examine written responses of participants and anticipate the possibility of recurrent suicidal thoughts. The accuracy of the NLP-based program in correctly elucidating suicidal thoughts was 61%. This can prove to be revolutionizing for psychiatric management as the risk of suicide can be determined among patients through their social media posts and text messages. Finally, Zohuri and Zadeh explored the utility of AI for mood analysis, depression detection, and suicide risk management [20]. They determined that the integration of optical character recognition with AI could result in the early identification of depression and the prevention of suicide. 

AI has shown potential in identifying psychiatric disorders and treatment responders by combining imaging data. ML has been able to predict major depressive disorder (MDD) patients by analyzing structural brain imaging data and envisaging those patients who respond to treatment with an accuracy of 89% [21]. In addition, in healthy adolescents, ML has been able to predict MDD with an accuracy of 70% [22]. By combining diffusion tensor imaging (DTI) data and neuropsychiatric measurements, ML is able to differentiate bipolar type 1 and bipolar type II and classify pediatric bipolar disorder from healthy volunteers with an accuracy of 79% [23,24]. Some studies have classified bipolar disorder and MDD patients with good accuracy of 92.1%, 79.3%, 74.3%, and 69.1% [25-28]. Three SVMs used 1.5T MRI scan data to differentiate schizophrenia and bipolar patients with an accuracy of 88%. However, the accuracy was comparatively less, approaching only 66% and 53% in healthy subjects and patients with bipolar disorder, respectively [29].

The potential utility and applications of AI have grown exponentially in various psychiatric illnesses [30]. AI has become an integral part of psychiatry, psychology, and psychotherapies [31]. AI has the potential to revolutionize healthcare. It has been used to diagnose conditions, predict mental health crises, and alert healthcare providers [32]. Overall, AI chatbots have been variably efficient in encouraging a healthy lifestyle, smoking cessation, treatment, and medication adherence, or decreasing drug abuse [33]. Integrating AI into mental health interventions has proven positive in chronic pain patients having comorbid depression and anxiety [34]. The use of conversational agents in mental health treatment has garnered attention as an innovative and cost-effective method to achieve higher success outcomes. Several studies have concluded that an AI-powered conversational agent can successfully deliver cognitive-behavioral therapy to patients with chronic pain complaining of depression and anxiety. Furthermore, patients with chronic pain have shown meaningful engagement and adherence with conversational agents as compared to the standard industry metrics [35]. Empirical evidence has shown that AI-enabled, empathetic, text-based conversational mobile mental well-being apps are extremely effective for people self-reporting depressive symptoms [36]. Psychiatry has been using deep learning methodologies involving ML to diagnose and treat depression. The accuracy of AI models is successfully predicted utilizing internal, external, and empirical validation. For internal validation, the dataset used to train the model is utilized, for external validation, a separate dataset beyond the study sample is utilized for generalisability, and for empirical validation, the AI model is implemented in real-life situations for practical demonstration. This underscores the crucial role of validation studies in deploying AI in mental healthcare [37]. Other studies have investigated the prospects of AI chatbots in peoples’ interaction with expressions of sadness and depressive behavior [38]. More studies have shown that chatbots have high user satisfaction ratings; hence, they may be used to alleviate anxiety and depression [39]. Additionally, research has suggested that the technical advancement of AI chatbots may have the potential to reduce depression symptoms and also increase patient engagement [38]. Moreover, some studies have recommended the use of chatbots to administer the Patient Health Questionnaire-9 (PHQ-9) to assess depressive symptoms interactively and remotely [40]. However, the efficacy of these chatbot versions and validation have not been investigated.

Utilizing AI in dementia and AD research is critical because it has assisted researchers in getting a dynamic approach in improving and understanding these conditions. Although various studies have shown a rising use of AI and machine learning advances in cognitive impairment and Alzheimer’s dementia, AI has been widely used to develop cognitive assessment among patients with mild cognitive impairment and Alzheimer’s dementia [41]. However, other studies have confirmed that artificial neural networks and conventional statistical practice can also help in diagnosing Alzheimer’s disease accurately [42]. Both of these AI programs have been used by professionals in the sector to aid in early detection and also to promote patient-centered outcomes. These developments demonstrate the significant impact of applying artificial intelligence technologies in dementia and Alzheimer’s disease research. Today, AI technologies have become predominant in cognitive assessment, early recognition of cognitive impairments, personalized therapy, and even in identifying persons who could potentially progress to dementia. Various studies reveal the role of AI in specific clinical tools development which could detect early symptoms of AD through eye movement data. It was a less invasive way of imaging research compared to traditional clinical tools [43]. In addition to that, AI was an inseparable part of the tool’s development, which allowed automated analysis of brain images in dementia patients [44]. This tool was able to significantly increase diagnostic capability and with the combination of neuroimaging, it is possible to identify some brain modifications at the early stage of cognitive decline to ensure better diagnostic and treatment outcomes. Thus, AI innovations and technologies significantly improve dementia and AD research, which will also positively affect patient results.

Schizophrenia research and clinical practice may similarly benefit from the use of AI and ML algorithms. SVMs, random forests, K-nearest neighbors, and deep learning have been employed to classify schizophrenia patients using morphological information obtained from brain regions such as the amygdala and hippocampus [45]. ML, for example, can distinguish between different subtypes of schizophrenia, such as positive and negative subtypes, based on neurobiological factors [46]. Considering the potential of AI and ML models, these are promising applications for the future of substance use disorders. For instance, a study confirmed the potential of these models in predicting and even screening substance users or non-users. ML models can successfully differentiate between substance users and controls based on the composition of the gut microbiota [47]. Therefore, AI and ML can be a powerful tool in the timely identification and treatment of substance use disorder. The utilization of ML models to predict treatment response in patients with cannabis use disorder is evidenced by recent trials in which they demonstrated high predictive ability, with an AUC value of more than 0.70 [48]. Many studies have explored the potential utilization of ML models in predicting the response to substance use disorder treatments. Other studies have compared the accuracy of logistic regression and random forest models in predicting individual variation in treatment response [49].

Table 1 summarizes the studies showing the validity of AI tools for diagnosing NPDs.

**Table 1:** Summary of studies showing validity of AI tools for diagnosing neuropsychiatric disorders. fMRI: functional magnetic resonance imaging; s MRI: structural magnetic resonance imaging; SVM: support vector machine; FoBA: forward and backward strategy; MDD: major depressive disorder; BPD: bipolar disorder; AI: artificial intelligence; SPECT: single photon emission computerized tomography; SUD: substance use disorder; TEDS-D: treatment episode dataset – discharges

Author (Year)	Data Type	AI Tool/Software Used	Number of Participants	Findings (Accuracy, specificity, Sensitivity)	Conclusion
Fei et al (20.21) [15]	Emotion Patterns	MobileNet, Support Vector Machine	61	73.3% Accuracy	The facial recognition algorithm can relatively accurately detect cognitive impairment
Kang et al. (2019) [16]	Neuropsychological Tests (NPTs)	TensorFlow	14,926	96.66% Accuracy	The artificial neural network can accurately detect cognitive impairment
Torres-Gaona et al. (2023) [9]	EEG	Supervised Machine Learning, MJN-SERAS (MJN Neuroserveis)	50	94.7% Sensitivity, 92.2% Specificity	AI Detection of epileptic seizures is sensitive and specific
Nadeem et al. (2016) [7]	Text-based Social Media posts	Twitter (Now X)	2.5 million	81% accuracy	Major Depressive Disorder (MDD) can be accurately predicted from social media
Singh et al. (2020) [18]	Facial Expressions	Adaptive moment estimation (ADAM)	150	67.6% accuracy	Computerized facial emotion detection has a relative accuracy
Cook et al. (2016) [19]	Text-based Assessment Answers	Natural Language Processing (NLP), Wired Informatics	1,453	73% positive predictive value 76% Sensitivity, 62% Specificity	The NLP-based machine learning model had a reasonably high predictive value
Foland-Ross et al. (2015) [22]	MRI	Support Vector Machine,	33	69.7% Accuracy 69.3% Sensitivity 70.0% Specificity	Identifiable differential brain structure could accurately predict the onset of depression withing 5 years of Brain analysis
Wu et al. (2016) [23]	Diffusion-weighted Imaging scans	Cambridge Neurocognitive Test Automated Battery (CANTAB)	70	76% Accuracy	The different phenotypes in bipolar disorder were accurately identified.
Mwangi et al. (2015) [24]	Diffusion-weighted Imaging scans	Support vector machines (SVM)	16	78.12% accuracy 68.75% Sensitivity 87.5% Specificity	The ability for the SVM to predict Pediatric Bipolar Disorder was significantly accurate.
Jie et al. (2015) [25]	MRI, fMRI	SVM-FoBa	21	92.07% Accuracy	SVM-FoBA was able to accurately distinguish between MDD and BPD.
Schnack et al. (2014) [29]	s MRI	Support vector machine (SVM)	136	61-76% accuracy	the SVM could accurately differentiate healthy vs schizophrenia, and bipolar vs schizophrenia, but was less accurate in differentiating health vs schizophrenia.
Inkster et al. (2018) [36]	Text-based Messaging	Wysa	129	67.7% found helpful.	AI Conversational therapy was found to be 67.7% helpful for participants struggling with mood disorders.
Chin et al. (2023) [38]	Text-Based Messaging	SimSimi	152,783	NA	It was found that people were more willing to discuss their emotional wellbeing with an AI Chat Bot versus other social media platforms.
Kalafatis et al. (2021) [41]	Text-Based Assessment Answers	Integrated Cognitive Assessment (ICA)	230	81-88% Accuracy	The AI Model was able to accurately detect cognitive impairment
Swietlik and Białowąs (2019) [42]	SPECT Cerebral Blood Flow Testing	Artificial Neural Network, TIBCO Software	132	93.8% Sensitivity 100% Specificity	The artificial neural network positively impacts Alzheimer disease diagnostics.
Guo et al. (2020) [45]	s MRI	FreeSurfer 6.0, support vector machines (SVM)	126	81.75% Accuracy	The SVM was able to accurately distinguish hippocampal and amygdaloid areas that had diagnostic value for schizophrenia
Liu (2023) [47]	Fecal Samples	Random Forest Classification	333	89.57% accuracy	Based on human host gut microbiota, the random forest classification was able to accurately diagnose drug abuse
Tomko et al. (2023) [48]	Urine Cannabinoid Tests	Multivariable Machine learning model	302	73% Accuracy	the multivariable/machine learning model was able to accurately predict which patients were going to respond to multi-component treatment for cannabis use disorder
Acion et al. (2017) [49]	SUD Outpatient Discharge information	TEDS-D 2006-2011, machine learning framework	99,013	79.3-82.0% Accuracy	The machine learning framework was able to accurately determine patients who would be successful in the substance use disorder treatment.

Clinical reliability of AI tools

Clinical reliability, a term closely related to clinical validity, refers to the reproducibility of diagnostic metrics in calculating the same data across varying and randomized measurements. In the context of AI and neuropsychiatry, reliability refers to the ability of AI tools to output the same diagnosis, given the symptoms are consistently the same in randomized patients. Reliability is integral to AI diagnostic tools and must be measured since reliability dictates the choice of measures used for assessment in eligibility and exclusion while also being a dictator of quality control [50]. 

Various factors can drastically affect the reliability or generalizability of the use of AI tools. One crucial factor is the quality of input data that is used in the training of these tools. An aspect of neuropsychiatry that is widely used in diagnosis is neuroimaging data. Unreliable and lack of adequate neuroimaging data has been shown to contribute toward poor generalizability [51]. For example, when collecting functional MRI (fMRI) data specifically, the reliability of functional connectivity can be impacted by the patient’s arousal level, signal-to-noise (or artifact) correction ratio, and the amount of fMRI data collected [52,53]. Hence, training the AI tools with precise and valid inputs becomes crucial. Additionally, unlike the combination of “expert book knowledge” and patient-specific inputs that a clinician uses, AI tools may not fully capture heterogeneity in a patient’s symptoms. This means that to truly deliver patient-centered care, AI tools will need to have training inputs that encompass the dimensionality of symptoms as well as other ecological factors such as comorbid symptoms, medications, and lifestyle [53, 54]. 

Clinical reliability is the ability to consistently and stably provide diagnosis and treatment through evaluations, assessments, and measurements. Clinicians are required to be both clinically valid and clinically reliable to practice medicine, as such, if AI is to take a prominent role in diagnosis and treatment, it must also be clinically valid and reliable. While still in its earlier stages, preliminary testing with AI in neuropsychiatric care has overall suggested that AI has demonstrated consistent outcomes for patients with depression and anxiety in comparison to the standard psychiatric treatment options available [55]. This is promising to suggest that AI does have the potential for clinical validity as it advances through its use in practice. Additionally, AI has been tested in the form of experience sampling (ES), a form of measurement that takes primary symptoms from the Diagnostic and Statistical Manual of Mental Disorders, fifth edition (DSM-5) as well as neuroimaging findings from previously diagnosed patients to offer diagnosis to new patients through comparative data interpretation and showed promising results for identifying MDD as well as substance use disorders [2]. This suggests that AI is proving capable of providing consistent clinically valid diagnoses through ES, leading to promising future expectations of AI in neuropsychiatric care. 

To understand the importance of reliability in neuropsychiatry diagnosis, it is important to understand the complexity of psychiatry historically. Despite the efforts of the World Health Organization (WHO) and the American Psychiatry Association (APA) to develop psychiatric nomenclature and diagnostic criteria that paved a path toward defining psychiatric diagnosis accurately, reliability of psychiatric diagnoses remains the biggest challenge for mental health professionals. The conventional "open-form" approach for interviewing patients in terms of psychiatric disorders is not reliable. Having said that, it is evident from the research that clinicians with good interviewing skills can develop therapeutic rapport with patients [56]. 

There are many shortcomings currently particularly in the field of psychiatry in general such as the requirement of years-long training of clinicians and resources required for it, lack of objective tests, and reliance on the patients for subjective symptoms [56]. Historically, these needs have been tried to be met by devising rating scales and structured interviews which again have shortcomings in terms of clinicians' “individual impression” instead of using the same tools [57]. 

Subsequently, to solve this quest of complexities in the world of psychiatry, the use of AI has long been suggested. Surprisingly, a massive paradigm shift is just observed. AI and ML are being reported to have a massive potential for reliability on the different levels of making diagnoses and can also lead to the development of new diagnostic tools. Using ML can help identify patterns in brain imaging with a better understanding of the neurological basis of disorders. Patterns in the patient's history are of great importance in psychiatry and AI identifies those patterns better than average human clinicians who can overlook the minor details. AI can decode the complexity of mental disorders by using network models and studying temporal and causal dependencies among the symptoms of mental disorders [57]. Digital phenotyping can benefit in identifying risk factors pointing towards the worsening of the symptoms. Furthermore, AI is most cost-effective in this time of immense mental healthcare needs and constraints of financial resources in the field globally.

Without deviating from the topic, it is important to quote here the newly emerging field of ‘’precision psychiatry’’ in which advancements in AI are combined with numerous biomarkers and genetic loci associated with psychiatric disorders and relevant treatments are being discovered by employing neuroimaging and multiomics [58]. Not only that, it has significant applications in diagnosis prediction by studying newly emerging data science methodologies. Studies have shown high predictive outcomes for patients with AD, autism spectrum disorder (ASD), and schizophrenia.

Challenges and limitations

As previously mentioned, AI primarily uses ML algorithms that utilize various sources such as statistics, computer sciences, linguistics, and concepts of deep learning to generate results [59-61]. Although these computational tools may be used as a powerful early detection and screening platform for neuropsychiatric disorders in the near future, the current landscape of AI presents several limitations, which pose significant challenges that require careful consideration.

AI has been increasingly used in neuropsychiatry to predict disease onset. A promising study suggested that the AI algorithm could find cardiovascular disease with an accuracy of 70%, which is comparable to existing risk prediction methods such as screening blood cholesterol levels [62]. However, the crucial question is whether we can blindly trust the use of AI models in clinical settings. AI has been thought of as a solution to many problems in healthcare settings and beyond. Despite promising results, AI does not have human intelligence, common sense, intelligence of science fiction, and a high level of reasoning [62-64]. There is a noticeable scarcity of comprehensive data, indicating a requirement for substantial research to enhance current AI practices. Unlike other medical fields, neuropsychiatry lags in the application of AI tools, primarily due to a lack of focused research. Hence, only with the input of additional data that is accurate and clinically relevant will the reliability of AI tools improve. However, there is optimism about the potential applications of AI in neuropsychiatry, and efforts are being made to bridge the gap and improve the reliability of AI tools.

Another limitation of AI that has been set forth by researchers and clinicians is that AI applications could potentially lose capacity and insight during clinical decision-making. Therefore, while AI can play a crucial role in identifying early signs and symptoms of neuropsychiatric disorders, AI algorithms require careful monitoring and supervision for the care and treatment of vulnerable patients. Moreover, with the quickly emerging advancements in technology, there is an increasing risk of hacking and leakage of patient data. The intricate nature of patient capacity, which can fluctuate over time, heightens ethical complexities. This dynamic aspect often leads to increased skepticism and mistrust of AI-enabled tools in neuropsychiatric settings. Another significant challenge of AI usage is the deficiency in legal frameworks to safeguard patient privacy in the face of widespread AI usage [64]. Existing laws fall short of addressing the rapid advancements in AI technology, necessitating a reevaluation of regulations to ensure robust data protection. Hence, the applications and software that are being utilized as early-detection tools would require regular updates and optimization to maintain user security and most importantly, keep personal health information safe and secure. 

While there has been significant improvement in the validation of AI tools, there are still many factors that contribute to doubt amongst physicians regarding the safe use of AI in clinical settings. Some key points of concern are dataset shift and data quality issues, fragility of algorithms, back-box opacity, software errors, system context, and complexity [64]. In certain cases, it could be even more challenging to validate AI compared to traditional statistical models as the results and algorithms of the AI model could potentially change with repeated use [65]. Furthermore, while AI is gaining prominence, it is crucial to acknowledge its limitations, especially in replicating human empathy. The therapeutic relationship in psychiatry heavily relies on human connection, an element that current AI technology struggles to reproduce effectively. Hence, the reproducibility of healthcare-based AI usage is particularly poor as only 23% of studies from 2017 to 2019 have utilized AI and ML datasets to obtain results [66].

Technical errors are inherent in AI systems, and complete dependence on them remains a distant goal. Ongoing research aims to mitigate lapses and glitches in AI-enabled tools, fostering increased trust in the technology. Language limitations also pose a challenge, as the majority of AI research and applications are in English. This linguistic bias excludes a significant portion of the global population. The accessibility of AI-enabled tools is subsequently hindered by social and economic factors as well. The initial stages of AI application come with a cost, limiting access for everyone worldwide. Overcoming these challenges requires time, education, and a shift in societal attitudes toward embracing and trusting AI technologies. With this in mind, there is a crucial need for robust guidelines which are currently not present in the United States or the European Union for appropriate standardization of AI products [67,68]. As guidelines and technology advance, it is our hope that these challenges will be surmounted, allowing for the full realization of the benefits of AI-enabled tools in neuropsychiatry. 

The confidence of physicians (specifically psychiatrists) in using clinical AI models varies significantly. In a global survey of 791 psychiatrists, 17% believed that computers could provide equivalent empathetic care as a human. In contrast, 75% are of the opinion that computers will be just helpful for documentation purposes [67]. Therefore, while there has been tremendous growth and innovation in the use of AI in neuropsychiatry, it is unlikely to fully replace the role of psychiatrists in clinical settings due to the field’s irreplaceable reliance on empathy, understanding, and patient-physician interaction [67]. More importantly, due to technology's current and future limitations, the use of AI in medicine must be done responsibly [62,63,69].

Future directions

While AI is unlikely to fully replace the role of psychiatrists in clinical settings, with the introduction of more robust data, guidelines, and validation technology in clinical practice, AI can serve a supporting role in the decision-making process for the care of patients. Specifically, with the introduction of telehealth platforms, AI and ML can increase the accessibility of healthcare for patients in medically underserved settings. Research efforts in this arena have already begun with startups such as the Davos Alzheimer’s Collaborative (DAC). The DAC is currently using neuroimaging and deep learning AI tools coupled with smartphone technology to monitor cognitive aging and biomarkers of AD (and other forms of dementia) in vulnerable patient populations all around the world [70]. Studies such as the ones being conducted by the DAC have shown promising potential in developing practical and easy-to-implement methods of preventive care for devastating diseases. However, due to the current and future limitations of technology, the use of AI in medicine must be done responsibly [64,69]. AI, while a rising and promising tool for diagnosis and decision-making, still must rely on electricity and technology to be effective. If something were to happen, such as a power outage, AI cannot be effective. As such, it is important to outsource to AI minimally, while still maintaining psychiatric training for decision-making and diagnosis. Additionally, medicine is not a black-and-white field. AI can be beneficial for the more cut-and-dry cases seen on occasion but it has a long way to go when it comes to more complex cases. AI, where it currently stands, is incapable of overcoming “Fuzzy Logic” where the information provided does not fit into specific black-and-white boxes [3]. If we were to fully integrate AI in its current state, it could risk the reliability of diagnostics on more complex neuropsychiatric cases, such as complex post-traumatic stress disorder (CPTSD). In order to prove that AI has the capacity to handle more complex cases, it would need to be studied more extensively than it has been currently and trained on how to view diagnostics on a sliding degree scale rather than just in a black-and-white format [65]. 

This current lack of research does not mean AI cannot excel in neuropsychiatry. If undertaken with proper considerations for fail-safes and appropriate research conduction, implementation of AI into clinical practice could be beneficial. Implementing AI into diagnostic strategies could allow physicians more time to care for their patients' conditions, rather than spending their time strictly diagnosing. Additionally, technology is being implemented where AI can assess a patient's mood and behaviors through observation, leading to a potentially more accurate baseline diagnosis and understanding of a patient's current mental state [71]. This has the potential, if done appropriately, to revolutionize the way that crisis care is implemented and identified, as it can help assess who is in an active crisis or who is starting to struggle with their mental health. 

## Conclusions

Advancement of technology in the field of healthcare, especially in AI models, can make significant contributions in clinical settings. AI has shown promising results and can be implemented throughout patient care, which can aid in reaching accurate diagnoses and devise individualized management plans. This is absolutely vital for neuropsychiatric conditions as it is difficult for healthcare providers to accurately predict certain subjective findings including patient emotions and pain.

Research has revealed that deep learning and ML offer innovative methods to classify patients, predict outcomes, and provide support. However, it is crucial to develop guidelines to ensure that ethical standards are maintained, and the technology is used effectively for favorable patient outcomes. Ongoing research can help us develop AI’s full potential, improve the quality of care, and ultimately save lives.
